# Nuclear iASPP determines cell fate by selectively inhibiting either p53 or NF-κB

**DOI:** 10.1038/s41420-021-00582-1

**Published:** 2021-07-26

**Authors:** Wenjie Ge, Yudong Wang, Shanliang Zheng, Dong Zhao, Xingwen Wang, Xiaoshi Zhang, Ying Hu

**Affiliations:** 1 School of Life Science and Technology, Harbin Institute of Technology, Harbin, Heilongjiang Province 150001 China; 2 Department of Clinical Laboratory, Qilu Hospital of Shandong University, Jinan, Shandong 250012 China; 3 Shenzhen Graduate School of Harbin Institute of Technology, Shenzhen, 518055 China

**Keywords:** Cancer, Cell biology

## Abstract

p53 and NF-κBp65 are essential transcription factors (TFs) in the cellular response to stress. Two signaling systems can often be entwined together and generally produce opposing biological outcomes in a cell context-dependent manner. Inhibitor of apoptosis-stimulating protein of p53 (iASPP) has the potential to inhibit both p53 and NF-κBp65, yet how such activities of iASPP are integrated with cancer remains unknown. Here, we utilized different cell models with diverse p53/NF-κBp65 activities. An iASPP(295–828) mutant, which is exclusively located in the nucleus and has been shown to be essential for its inhibitory effects on p53/NF-κBp65, was used to investigate the functional interaction between iASPP and the two TFs. The results showed that iASPP inhibits apoptosis under conditions when p53 is activated, while it can also elicit a proapoptotic effect when NF-κBp65 alone is activated. Furthermore, we demonstrated that iASPP inhibited the transcriptional activity of p53/NF-κBp65, but with a preference toward p53, thereby producing an antiapoptotic outcome when both TFs were simultaneously activated. This may be due to stronger binding between p53 and iASPP than NF-κBp65 and iASPP. Overall, these findings provide important insights into how the activities of p53 and NF-κBp65 are modulated by iASPP. Despite being a well-known oncogene, iASPP may have a proapoptotic role, which will guide the development of iASPP-targeted therapies to reach optimal outcomes in the future.

## Introduction

Mammalian cells are unavoidably and continually affronted by various stresses. To maintain tissue homeostasis, a diverse range of mechanisms have been developed. The stress response contributes to cellular decision-making processes governing cell death and is thus essential in human diseases, including cancer.

p53 and NF-κBp65 are key regulators of stress signaling. p53 plays a critical role in tumor suppression. Genomic mutations of p53 exist in about 50% of human cancers [1]. In contrast, NF-κBp65 is often constitutively activated in cancers, highlighting the oncogenic identity of this gene [2]. Both proteins normally remain inactive under basal and unstressed conditions. Upon a variety of stresses, such as DNA damage [3], hypoxia [4], and oncogene activation [5], p53 is intensively modified. Some of the modifications can disrupt its interaction with E3 ligase MDM2 (murine double minute 2e), the principal cellular antagonist of p53, and protect p53 from proteasome-mediated degradation [3, 5, 6]. Stabilized p53 is translocated to the nucleus, where it transactivates target gene expression, leading to cell death, senescence, or metabolic reprograming dependent on the stress type, duration, and strength [7–9]. Cells with defective p53 not only promote carcinogenesis but also confer resistance to chemotherapeutic drug-induced cell death [10]. Stimuli such as viruses, cytokines, DNA damage, or antigen receptors can induce the activation of NF-κBp65 [11]. This is caused predominately by the phosphorylation and subsequent degradation of its cytosolic binding partner and inhibitor IκB (Inhibitor of kappaB) [12]. Once free of IκB binding, NF-κB translocates to the nucleus and functions as a transcription factor (TF) to promote the expression of genes that promote survival, proliferation, or inflammation, in turn favoring tumor development and chemoresistance [13–15].

Given the immense importance that the p53 and NF-κB pathways have in cellular physiology and pathology, it is not surprising that cross talk between these two transcriptional regulatory networks has been intensively studied. It is believed that the activation of NF-κB generally inhibits p53’s function and vice versa [16, 17]. However, NF-κBp65 has also been suggested to be required for p53-dependent apoptosis [18] and NF-κBp65 has been shown to activate p53 in inflammatory responses. Clearly, signaling mediated by p53 and NF-κB is often intertwined, leading to synergistic or antagonistic effects in a cellular context- and stimuli-dependent manner. Thus, unveiling how p53 and NF-κB are coordinately regulated is critically important, because it may help to identify and develop effective strategies to modulate cell fate, particularly in the context of tailored anticancer therapies.

A number of studies have provided evidence of selective activation of p53 or NF-κBp65. For example, the activation of Akt phosphorylates MDM2, leading to nuclear translocation of MDM2 and subsequent degradation of p53 in the nucleus [19]. Akt can also phosphorylate IKKB (inhibitor of nuclear factor-kB kinase subunit beta), which activates signals leading to the degradation of IκB and the activation of NF-κBp65 [20]. In contrast, the tumor suppressor ARF (ADP-ribosylation factor) binds with MDM2 and disrupts its inhibitory effect on p53 [5], on the one hand; while promoting CHK1(checkpoint kinase1)-mediated NF-κBp65 phosphorylation, inducing NF-κBp65 nuclear translocation and activation [21], on the other. In addition, both p53 and NF-κBp65 have been shown to be acetylated by p300/CBP (CREB binding protein) [22, 23]. Such modification is essential for the activity of both TFs. It has been shown that the pool of p300/CBP in the nucleus is limited [24] and that the activities of p53 and NF-κBp65 can be determined by their competitive binding with p300/CBP [25]. IκB represents another direct contact between p53 and NF-κBp65 that can bind and recruit both factors in the cytoplasm, thus inhibiting their transcriptional activities in the nucleus [26]. Given the critical roles of the functional interaction between p53 and NF-κBp65, cells may use other points of contact to regulate the reciprocal relationship of p53 and NF-κBp65.

Of note, an inhibitor of apoptosis-stimulating protein of p53 (iASPP), an oncogene that was initially identified as an NF-κBp65-binding partner and transcriptional inhibitor, has also been documented to bind with p53 and inhibit p53-dependent apoptosis in the nucleus. However, it remains unclear whether iASPP contributes to the cross talk between NF-κBp65 and p53 signaling [27, 28]. Recently, we demonstrated that iASPP can be catalyzed by caspase-3 after triggering apoptosis. The resultant iASPP(295–828) fragment is stable and translocates from the cytoplasm to the nucleus, where it exhibits better binding toward both p53 and NF-κBp65 and also has a more dramatic inhibitory effect on their transcriptional activities than full-length (FL) iASPP [29]. Nonetheless, it remains unclear how iASPP integrates its inhibitory effects on p53 and NF-κBp65, which generally lead to opposing biological outcomes. Here, we report that iASPP(295–828) simultaneously inhibits p53 and NF-κBp65 activity, thus enhancing NF-κBp65-regulated apoptosis but blocking p53-dependent apoptosis. We also describe that iASPP(295–828) has an intrinsic binding preference for p53, suggesting that it may contribute to the selective activation observed between p53 and NF-κBp65 under stress.

## Results

### iASPP(295–828) acts as an apoptotic inhibitor by blocking the transcriptional activity of p53

**Fig. 1:**
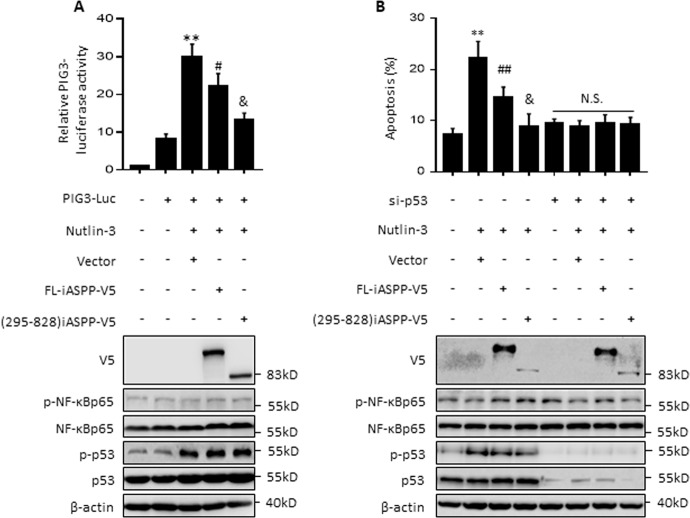
iASPP fragment inhibits apoptosis in MCF-7 when p53 was activated by Nutlin-3. PIG3 luciferase activity (**A**) and apoptosis levels (**B**) were determined by the luciferase reporter assay and Annexin V-FITC and PI staining in MCF-7 cells after the indicated treatments. The PIG3 luciferase activity in cells transfected with the same amount of empty vector plasmid was normalized to 1. Relative protein levels were determined by western blot. β-actin was used as an immunoblots loading control. **p < 0.01, relative to the untreated control; #*p* < 0.05 relative to the Nutlin-3-treated control; &*p* < 0.05 relative to Nutlin-3-treated FL control. N.S. not significant.

To understand the biological outcomes of nuclear iASPP activity in regulating p53 and NF-κBp65, we first examined the functional interaction between iASPP and p53 under conditions when p53 was activated but NF-κBp65 was inactivated. As shown, p53 was activated (indicated by increased p53 levels and PIG3 (p53 inducible protein 3) luciferase activity) after treatment of p53-wild type MCF-7 cells with the MDM2 inhibitor Nutlin-3; however, treatment had no obvious effect on the expression levels of NF-κBp65 or NF-κBp65 activation, as indicated by p-NF-κBp65 (Fig. 1A). Both FL-iASPP and iASPP(295–828) inhibited PIG3 luciferase activity, although the effect of iASPP(295–828) was more pronounced than that of FL-iASPP, with FL-iASPP reducing activity to 75% of the control and iASPP(295–828) to 40% (Fig. 1A).

We next used Annexin V and propidium iodide (PI) double staining to analyze apoptotic percentages under the same conditions. Nutlin-3 induced apoptosis by about 2.8-fold, while iASPP(295–828) reduced the effect of Nutlin-3 by about 60% (*p* < 0.05, Fig. 1B). Small interfering RNA (siRNA) specifically targeting p53 inhibited p53 expression, as confirmed by western blot. Accordingly, it completely abrogated Nutlin-3-induced apoptosis, as expected. Remarkably, both FL-iASPP and iASPP(295–828) lost their abilities to inhibit apoptosis after p53 knockdown (Fig. 1B). Consistently, we found that 5-FU induced mild levels of apoptosis in HEK293 cells and that p53 overexpression increased 5-FU-induced apoptosis levels. Additional ectopic expression of FL-iASPP or iASPP(295–828) inhibited p53-elevated apoptosis, and the effect was more evident with iASPP(295–828) than FL-iASPP (Fig. S1). These data suggest that iASPP(295–828) can inhibit apoptosis more efficiently than FL-iASPP by inhibiting p53’s transcriptional activity under conditions when p53 is activated and NF-κBp65 remains inactive.

### iASPP(295–828) has the ability to promote apoptosis by inhibiting the transcriptional activity of NF-κBp65

**Fig. 2:**
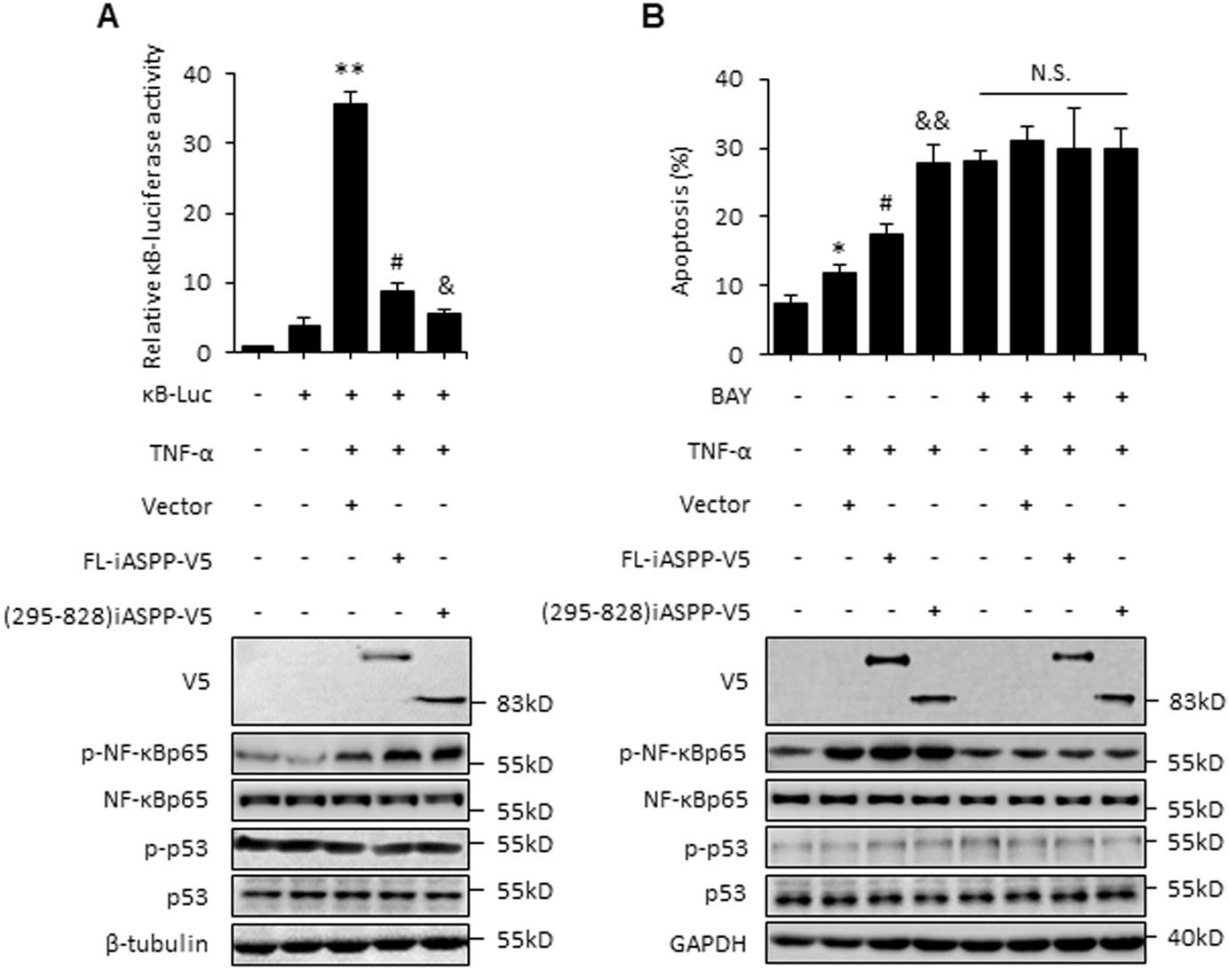
iASPP fragment promotes apoptosis in MCF-7 when NF-κBp65 was activated by TNF-α. MCF-7 cells were transfected as indicated and treated with or without 10 nM TNF-α in the presence or absence of 10 μM BAY117082 (BAY), then the transcriptional activity of NF-κBp65, as indicated by κB luciferase activity (**A**) or apoptosis levels (**B**), were measured. The κB luciferase activity in cells transfected with the same amount of empty vector plasmid was normalized to 1. Relative protein levels were determined by western blot. β-tubulin was used as an immunoblots loading control. *p < 0.05,**p < 0.01, relative to the untreated control; #*p* < 0.05 relative to the TNF-α-treated control; &*p* < 0.05, &&*p* < 0.01 relative to TNF-α-treated FL control. N.S. not significant.

iASPP was first identified as being able to bind to and inhibit an NF-κBp65 subunit; however, the biological significance of this interaction in the context of apoptotic regulation is not yet known. Given the observed increased inhibition caused by iASPP(295–828), we further investigated the impact of iASPP(295–828) on apoptosis in the context of NF-κBp65 activation. To this end, TNF-α (tumor necrosis factor-α) was applied to activate NF-κBp65 in MCF-7 cells [30]. The results showed that TNF-α activated NF-κBp65, as indicated by κB-luciferase activity and p-NFκBp65 levels, which had no obvious impact on p53 (Fig. 2A). iASPP and iASPP(295–828) reduced κB-luciferase activity by about 85%, which was more efficient than the effect of FL-iASPP (Fig. 2A). Also notably, FL-iASPP *tans*-localized into the nucleus after TNF-α treatment (Fig. S2A), which suggests that nuclear localization may be required for iASPP to inhibit NF-κB. TNF-α produced no obvious effect on cell proliferation, as indicated by BrdU incorporation rate (Fig. S2B). Inhibiting NF-κB activity by BAY117082 (BAY) failed to influences cell proliferation either (Fig. S2B). However, TNF-α induced mild levels of apoptosis. Interestingly, ectopic FL-iASPP expression promoted apoptosis by about 2.1-fold and similar levels of ectopically expressed iASPP(295–828) elicited a more dramatic proapoptotic effect in the presence of TNF-α (*p* < 0.01) (Fig. 2B).

To further investigate whether the proapoptotic effects of iASPP and its mutant were due to their ability to inhibit NF-κBp65, we examined the effects of iASPP in the presence of the NF-κBp65 inhibitor BAY. As shown, the p-NF-κBp65 protein was repressed by BAY, and BAY completely blocked the proapoptotic effect of iASPP in TNF-α-treated cells (Fig. 2B). In addition, in contrast to p53, NF-κBp65 overexpression abrogated 5-FU-induced apoptosis in HEK293 cells. Overexpression of FL-iASPP had no obvious impact on NF-κBp65-inhibited apoptosis, while iASPP(295–828) completely abrogated the effect of NF-κBp65 (Fig. S3A). These data suggest that iASPP(295–828) also exhibits proapoptotic activity by inhibiting the activity of NF-κBp65 under conditions when NF-κBp65 is activated.

**Fig. 3:**
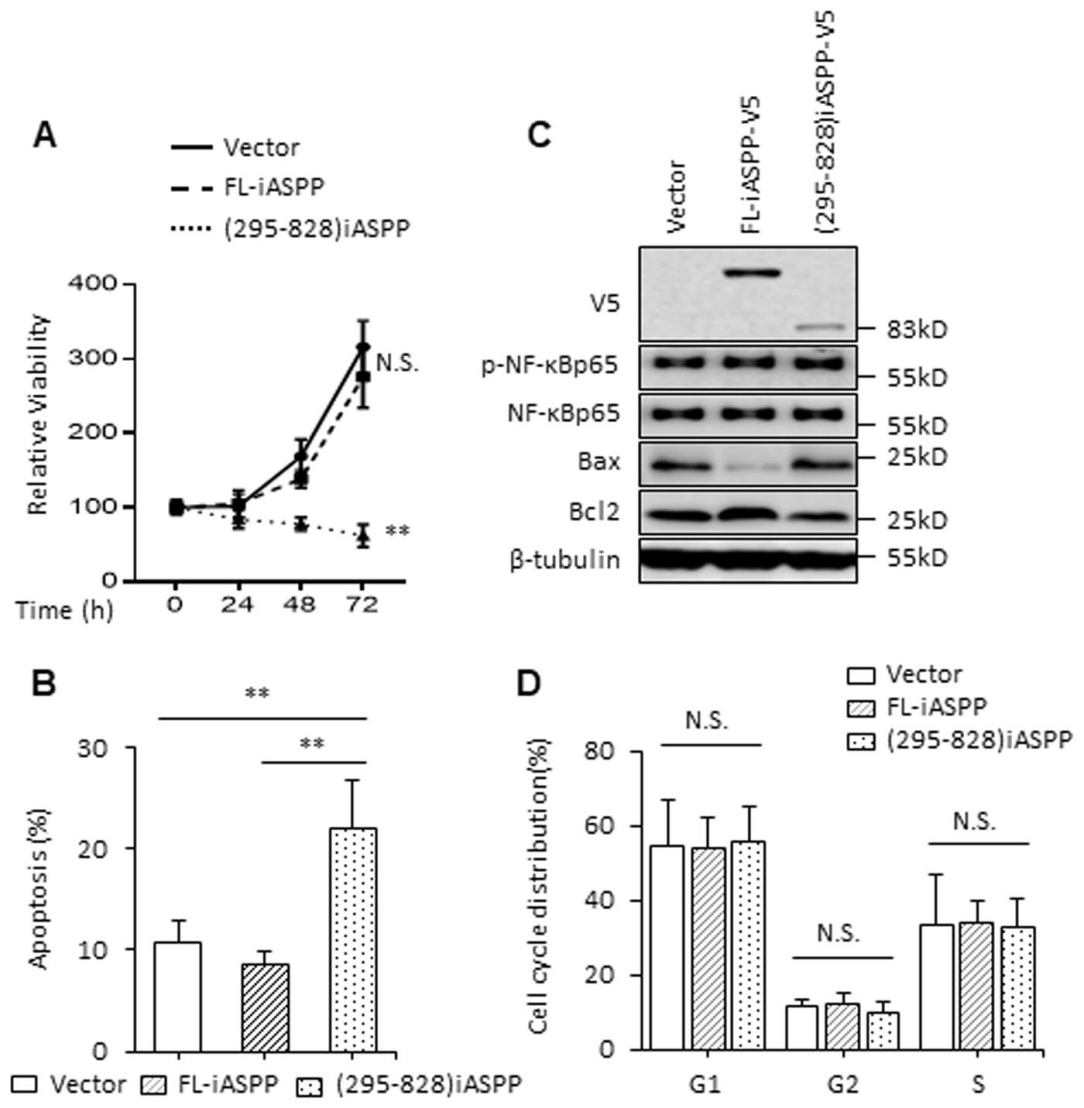
The cleaved iASPP fragment promotes apoptosis in NF-κBp65 constitutively activated SK-BR3. **A** SK-BR3 cells were transfected with Vector, FL-iASPP, and (295–828) iASPP plasmids, then cell viability was revealed by MTT assay. **p < 0.01, N.S. not significant. **B** Apoptosis levels were determined by Annexin V-FITC and PI staining in SK-BR3 cells after the indicated treatments. **p < 0.01. **C** Bax, Bcl-2, p-NF-κBp65, and NF-κBp65 were determined by western blot in SK-BR3 cells. β-tubulin was used as an immunoblots loading control. **D** Cell cycle arrest was revealed by PI staining in SK-BR3 cells after the indicated treatments. N.S. not significant.

**Fig. 4:**
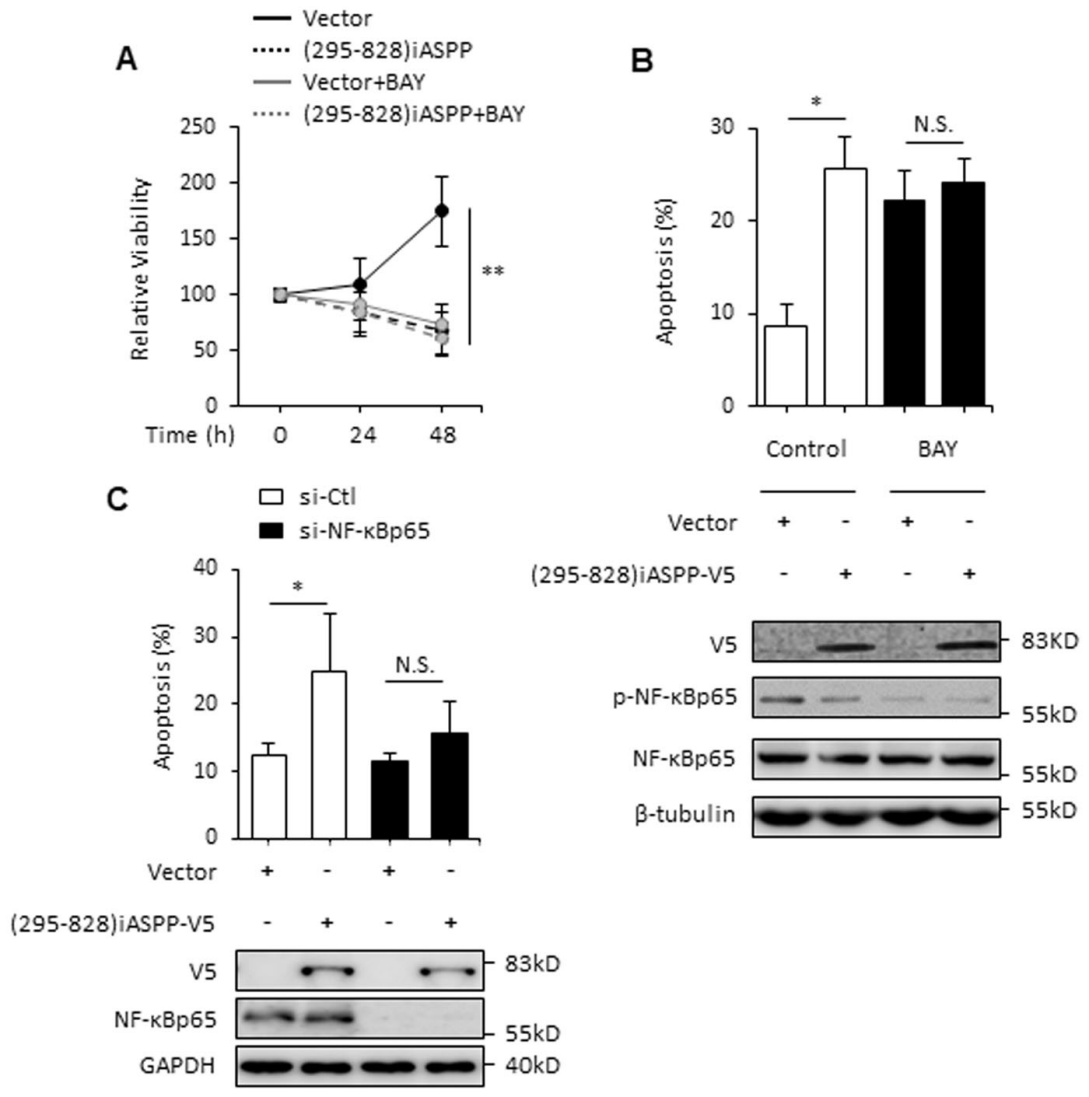
iASPP fragment promotes apoptosis through NF-κBp65 in SK-BR3. **A** SK-BR3 cells were transfected as indicated and treated with or without 10 μM BAY, then cell viability was revealed by MTT assay at 0, 24, 48 h. **p < 0.01. **B**, **C** Apoptosis was determined by Annexin V-FITC and PI staining and immunoblots in SK-BR3 cells after the indicated treatments. GAPDH and β-tubulin were used as immunoblot loading controls. *p < 0.05, N.S. not significant.

To further validate the findings described above, the regulatory effects of iASPP(295–828) on apoptosis were also examined in the breast cancer cell line SK-BR3, in which NF-κBp65 has been reported to be constitutively active and p53 has been inactivated by mutation [31, 32]. Interestingly, iASPP(295–828) alone significantly reduced the cellular viability of SK-BR3 cells in a time-dependent manner after transfection (Fig. 3A). This was mainly due to increased apoptosis because the proportions of Annexin V/PI-positive cells were markedly increased (Fig. 3B). Proapoptotic Bax (Bcl-2 associated X) was elevated and antiapoptotic Bcl-2 (B-cell lymphoma-2) was repressed (Fig. 3C). However, the cell cycle distribution was not disrupted under the above conditions (Fig. 3D). Importantly, this effect of iASPP(295−828) on cell viability and apoptosis was found to be completely abolished by either BAY (Fig. 4A, B) or siRNA-mediated NF-κBp65 knockdown (Fig. 4C). By contrast, FL-iASPP had no obvious impact on cellular viability. Subcellular fractionation assay revealed that FL-iASPP was predominately localized in the cytoplasm under basal conditions (Fig. S3B), suggesting that nuclear localization may be required for its activity to inhibit NF-κBp65.

**Fig. 5:**
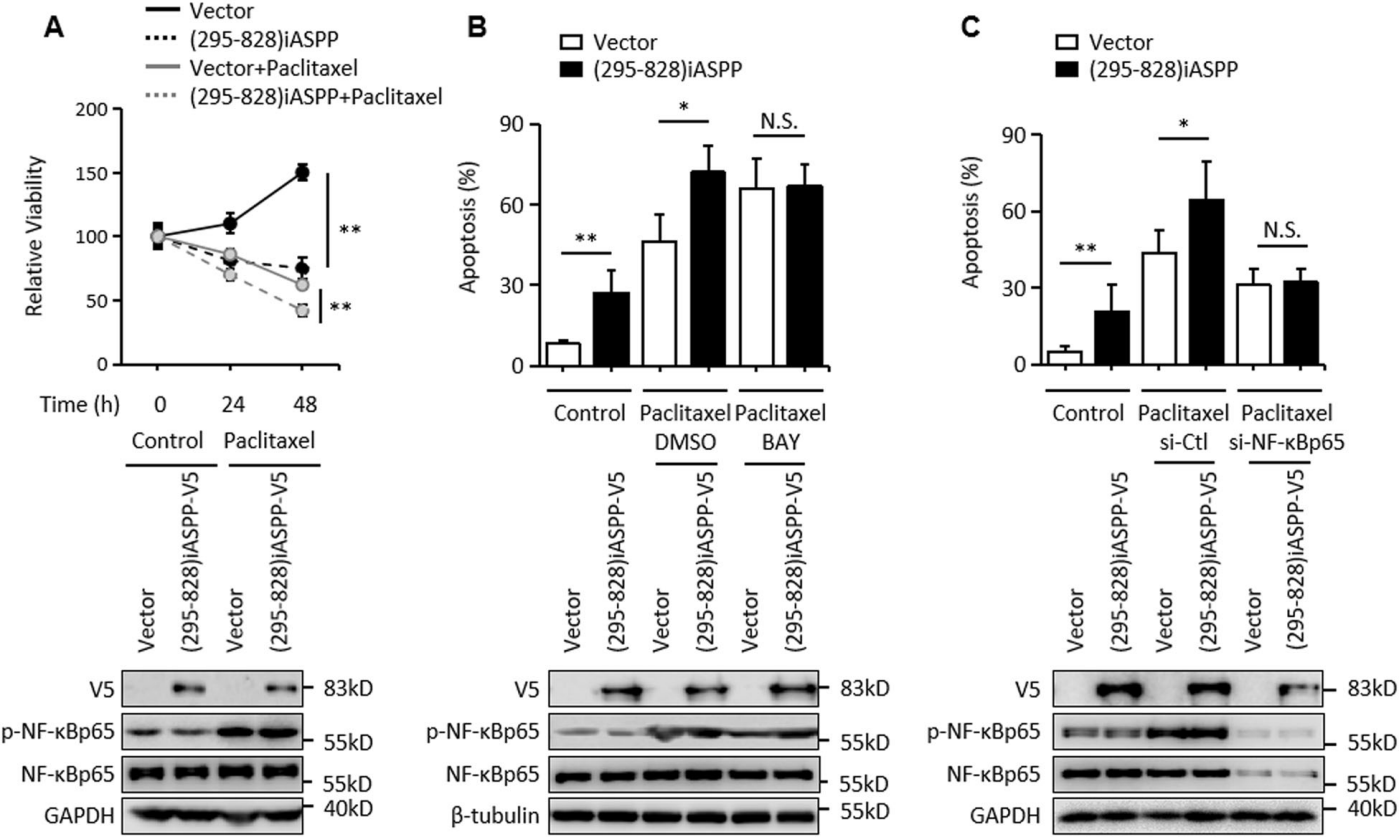
NF-κBp65 is required for iASPP fragment enhanced paclitaxel sensitivity in SK-BR3. SK-BR3 cells were transfected as indicated and treated with or without 5 μM paclitaxel, then cell viability (**A**) or apoptosis levels (**B**, **C**) were revealed by MTT assay or Annexin V-FITC and PI staining. Relative protein levels were determined by western blot. GAPDH and β-tubulin were used as an immunoblots loading control. *p < 0.05, **p < 0.01, N.S. not significant.

Moreover, SK-BR3 cells are well known to be highly resistant to chemotherapeutic agents such as paclitaxel [33]. Constitutively active NF-κBp65 may contribute to the resistance phenotype [34]. Given the fact that iASPP(295–828) is able to inhibit NF-κBp65, we speculated that iASPP(295–828) may sensitize cells to paclitaxel-induced apoptosis. Indeed, iASPP(295–828) markedly reduced cell viability after paclitaxel treatment (Fig. 5A). BAY or si- NF-κBp65 also increased paclitaxel-induced apoptosis, while no further effect was detected with a combination of iASPP(295–828) overexpression and BAY/ si- NF-κBp65 (Fig. 5B, C), suggesting that iASPP(295–828) sensitizes cells to paclitaxel-induced apoptosis by inhibiting the activity of NF-κBp65.

Taken together, the nuclear iASPP mutant iASPP(295–828) has the potential to promote apoptosis by inhibiting NF-κBp65 under conditions where NF-κBp65 is active and p53 is inactive.

### iASPP(295–828) preferentially inhibits p53 over NF-κBp65

**Fig. 6:**
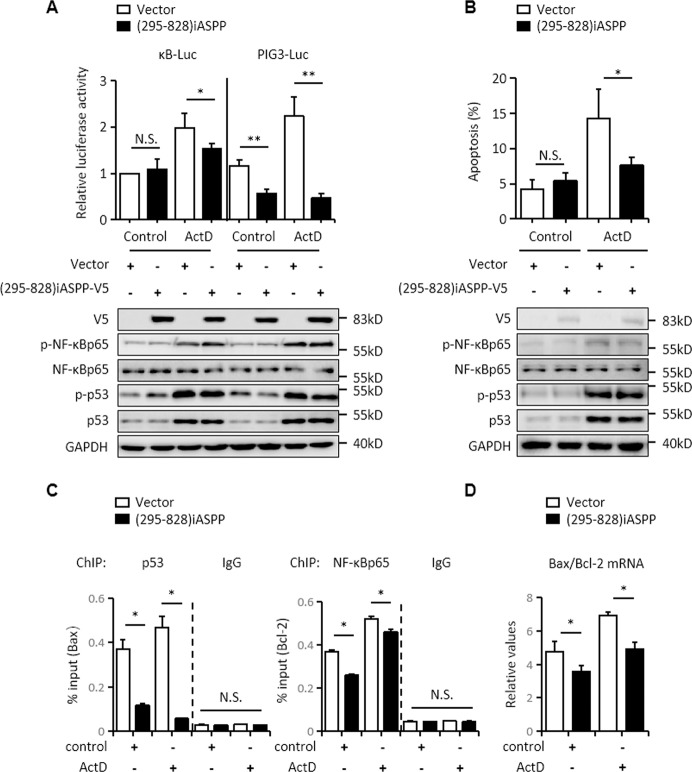
iASPP(295–828) preferentially inhibits p53 over NF-κBp65. **A**, **B** NF-κBp65 and p53 transcriptional activities, as indicated by κB and PIG3 luciferase activity, were measured by a luciferase reporter assay (**A**) and apoptosis levels were determined by Annexin V-FITC and PI staining (**B**) in RKO cells after the indicated treatments. The transfection efficiency and p-p53 and p-NF-κBp65 activation were determined by immunoblots. GAPDH was used as an immunoblots loading control. ActD actinomycin D. *p < 0.05, **p < 0.01, N.S. not significant. **C**, **D** RKO cells were transfected as indicated and treated with or without 7.5 nM ActD, ChIP assay was used to detect the binding of p53 and NF-κBp65 with Bax and Bcl-2 promoter (**C**). The mRNA levels of Bax and Bcl-2 were determined by qRT-PCR. GAPDH was used as a loading control (**D**). *p < 0.05, N.S. not significant.

Based on the fact that iASPP(295–828) acts as a double-edged sword in regulating apoptosis, by inhibiting either p53 or NF-κBp65, we next investigated whether iASPP(295–828) preferentially regulates either of these two targets. For this purpose, we utilized a model where both TFs were simultaneously activated by treating RKO cells with actinomycin D (ActD) [18]. As shown, p53 and NF-κBp65 were both activated, as indicated by the luciferase reporter assay or elevated p-NF-κBp65 and p-p53 expression (Fig. 6A, B). Interestingly, when the transcriptional activities of p53 and NF-κBp65 were elevated by ActD to similar extents (twofold), iASPP(295–828) reduced κB-luciferase activity to about 83% of the control, while PIG3 luciferase activity was reduced to ~19% of the control (*p* < 0.01, Fig. 6A). The preferential p53 inhibitory activity of iASPP(295–828) was also observed in ActD-treated HCT116 cells, suggesting it is a cell-type-specific event (Fig. S4A, B). In keeping with the above data, iASPP(295–828) inhibited apoptosis by about 50% in ActD-treated RKO cells (*p* < 0.05, Fig. 6B).

A ChIP assay revealed that the binding of p53 with the promoter region of its proapoptotic target Bax was reduced after iASPP(295–828) overexpression by approximately threefold under unstressed conditions and by eightfold under ActD-treated conditions (Fig. 6C). In contrast, the binding of NF-κBp65 and its antiapoptotic target Bcl-2 was reduced to a much lesser extent. These data were further validated by the mRNA expression of these targets as detected by real-time RT-PCR (Fig. 6D). These results indicate that iASPP(295–828) binds p53 more effectively than NF-κBp65 and thus has a more dramatic effect on p53.

### iASPP(295–828) binds with p53 more efficiently than with NF-κBp65

It has been proposed before that preferential binding of p300 may contribute to the selective activation of p53 and NF-κBp65. We asked whether iASPP prefers to inhibit p53 than NF-κB by modulating p300; however, this is unlikely to be the case, because p300 was not detected in RKO cells (Fig. S5A), in which iASPP was found to inhibit p53 than NF-κBp65 more efficiently (Fig. 6C, D).

**Fig. 7:**
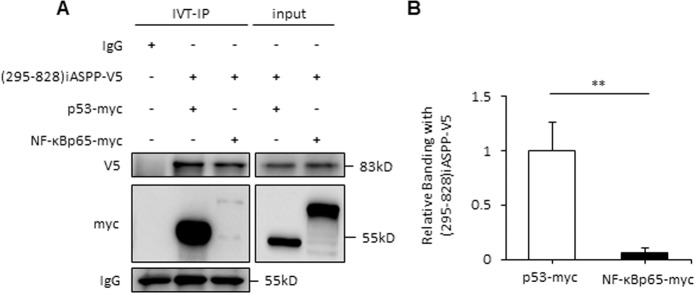
iASPP(295–828) binds with p53 more efficiently than with NF-κBp65. The interaction between (295–828)iASPP and p53 and NF-κBp65 were determined by a co-IP assay using in vitro-translated (295–828)iASPP-v5, p53-myc, and NF-κBp65 (**A**). The bar graph represents the relative p53 or NF-κBp65 proteins that bound with (295–828)iASPP (**B**). **p < 0.01.

We and others previously showed that iASPP/iASPP(295–828) inhibits p53 or NF-κBp65 activity by direct protein–protein interaction. Given that iASPP inhibits p53 relatively efficiently, we reasoned that iASPP may bind with p53 and NF-κBp65 differently. To test this hypothesis, an in vitro immunoprecipitation assay was conducted. Plasmid constructs expressing either p53 or NF-κBp65 were generated, both with a myc tag. The in vitro-synthesized p53 and NF-κBp65 proteins were monitored by western blotting using an anti-myc antibody. Similar levels of p53 and NF-κBp65 proteins were mixed with the same amount of in vitro-translated iASPP-v5 protein. iASPP was precipitated with an anti-v5 antibody. Co-precipitated p53 or NF-κBp65 were detected using an anti-myc antibody. The results showed that co-precipitated p53-myc was much more prevalent than NF-κBp65-myc (eightfold, *p* < 0.01, Fig. 7A, B). Therefore, we concluded that iASPP binds with p53 better than with NF-κBp65.

Recently, we reported that FL-iASPP translocates to the nucleus during cellular senescence; meanwhile, p53 and NF-κBp65 were simultaneously activated [35]. Under such conditions, we noted that iASPP’s ability to inhibit p21-luciferase reporter activity (a well-known p53 target) more efficiently than that of kB-luciferase reporter activity (Fig. S5B–D). In addition, the levels of an 80 kDa iASPP were increased in colon cancer tissues compared with the normal controls (Fig. S5E). These data are in line with our data shown above.

## Discussion

p53 and NF-κBp65 are central to the cell’s response to a variety of stresses, are linked to multiple fundamental biological processes, and thus play essential roles in carcinogenesis and drug resistance [11, 36–38]. Complicated cross talk between p53 and NF-κBp65 mediates the balance between life and death, but relatively few studies have explored the molecular mechanisms involved. It would be logical to propose that key elements in their regulatory networks may contribute to the selective activation of p53 and NF-κBp65, such as p300/CBP and IκB [25, 26]. Here, we tested this idea and revealed that a previously identified p53 and NF-κBp65 inhibitor, iASPP, can produce an anti- or proapoptotic effect depending on which factor, p53 or NF-κBp65, is predominately activated in cells. In addition, iASPP favors binding with p53 and therefore has a more dramatic inhibitory effect on p53 than NF-κBp65 when both are active.

We recently demonstrated that iASPP is mainly distributed in the cytoplasm under unstressed conditions in the majority of cancer types, where it elicits oncogenic activity by stabilizing anti-oxidative Nrf2 in a p53-independent manner [39]. It has also been reported that iASPP can produce an inhibitory effect on p53 in the nucleus because it loses its ability to bind with p53 in the cytoplasm due to iASPP dimer formation, which covers both iASPP’s p53-binding motif and its nuclear translocation signal [40]. Thus, in order to better understand the functional interaction between p53 and iASPP, we have to define the conditions under which iASPP is retained in the nucleus. Of interest, we previously found that the activation of caspases facilitated the cleavage of iASPP during apoptosis. The stable iASPP(295–828) fragment is mainly distributed in the nucleus because of the loss of the N-terminal residues that mediate iASPP’s dimerization. Notably, in this nuclear iASPP(295–828) mutant, the sites that contribute to its binding with both p53 and NF-κBp65 are retained [29]. Indeed, our data revealed that iASPP(295–828) binds with both p53 and NF-κBp65 and inhibits their transcriptional activities. In addition, such activities of iASPP(295–828) were much stronger than those of FL-iASPP, supporting the idea that the nuclearly localized iASPP monomer is required for its inhibitory effect on p53. Nonetheless, the biological outcomes induced by iASPP(295–828) are not well understood. Findings from the present study show that iASPP, a well-established oncogene, is actually able to promote apoptosis through the inhibition of NF-κBp65 under certain conditions. In addition, apoptotic cancer cells contain iASPP(295–828)

NF-κBp65 can be activated by TNF-α and chemotherapeutic drugs, which is generally associated with apoptotic tolerance [41]. However, inhibition of NF-kB activation by expression of a dominant-negative mutant form of IκBa (IκBm), which is resistant to phosphorylation and degradation, is not always able to increase the sensitivity to cytotoxic treatment [26], suggesting that additional levels of regulation of NF-κBp65 also exist. iASPP has been shown to inhibit NF-κBp65 both in vivo and in vitro [42, 43]; however, its activity remains unknown in the context of apoptosis. Here, our data clearly show that iASPP promotes TNF-α- and paclitaxel-induced apoptosis via inhibition of NF-κBp65. In agreement with the proapoptotic function identified by us, Kramer and colleagues have also reported that iASPP can promote apoptosis by stabilizing p300/CBP [44]. It is known that there are many “double-edged sword” genes in the human genome. Our data and those of others suggest that iASPP may be one such gene. Thus, caution needs to be taken in the development of iASPP-targeted therapies, because cell context needs are taken into consideration.

In addition, we found that iASPP’s binding with p53 is better than its binding with NF-κBp65 in an in vitro assay, and thus facilitates an antiapoptotic outcome in response to activation of both p53 and NF-κB. p53 and NF-κB share similar binding sites on iASPP (the SH3 domain and ankyrin repeats) [28], and it will be interesting to investigate whether p53 has a greater binding affinity with iASPP and thus competitively blocks its binding with NF-κBp65. This model needs to be validated in future studies. Nonetheless, the potential difference in its binding with p53 and NF-κBp65 opens up opportunities for the design of specific inhibitors to block iASPP’s interaction with p53 and strengthen its interaction with NF-κBp65. Thus, modulation of iASPP may become a useful strategy for the effective combination of p53 activation and NF-κBp65 inhibition.

Furthermore, we have investigated nuclear iASPP activity via ectopic expression of an iASPP nuclear mutant and with particular stimuli. However, FL-iASPP has been found to localize in the nucleus under certain circumstances. For example, M. Lu et al. have shown that iASPP predominately resides in the nucleus in some melanoma cell lines and patient samples. This is mainly due to the constitutive activation of cyclinB1/CDK1(cyclin-dependent kinase 1), which phosphorylates iASPP at key residues that mediate iASPP dimer formation [40]. We have also found that low-dose sublethal DNA damaging agents can promote the nuclear translocation of iASPP [35]. Here, we provide a potential explanation for why iASPP mainly behaves as an oncogene under the above conditions. Our results also revealed that the nuclear-localized FL-iASPP inhibits p53 more efficiently than NF-kBp65, suggesting that nuclearly localized FL-iASPP’s behavior is similar to that of the nuclear mutant. In addition, 80 kDa iASPP fragment is produced in the apoptotic malignant cells [29]. We also detected an endogenous 80 kDa iASPP in human tissues by an anti-iASPP antibody. The identity and the biological functions of this iASPP fragment need to be explored in the future.

In summary, iASPP can act as either a pro- or antiapoptotic factor, depending on whether p53 or NF-κBp65 is activated in the nucleus. Nonetheless, its ability to bind to and inhibit p53 is much stronger than to NF-κBp65 in nature. This study provides important insights into the selective regulation of p53 and NF-κBp65, and also provides guidance for the future development of iASPP-targeting therapies.

## Materials and methods

### Cell culture

RKO cells were maintained in RPMI-1640 medium (Gibco) supplemented with 10% (v/v) fetal bovine serum (Biological Industries) and 2 mM l-glutamine. MCF-7 and SK-BR3 cells were maintained in DMEM with the same supplement as shown above. All cell lines were grown at 37 ˚C in the humidified incubator (Thermo Scientific) with 5% CO_2_. Cell lines were routinely tested to exclude mycoplasma contamination and characterized by Genetic Testing Biotechnology Corporation (Suzhou, China) using short tandem repeat markers.

### Small interfering RNA (siRNA) and plasmid transfection

siRNAs specifically targeting p53, NF-κBp65, and nonspecific si-control were synthesized by GenePharma company. The sequences were shown as follows (si-p53: 5′-GAAGAAAATTTCCGCAAAA-3′, si-NF-κBp65: 5′-CAUCAACUAUGAUGAGUUUCC-3′). Full-length iASPP was kindly provided by Professor Xin Lu, Oxford University. Full-length NF-κBp65 and p53 were sub-cloned into a pCDNA3-myc3 vector using appropriate restriction enzyme digests. Plasmids expressing iASPP fragment (295–828) was generated by using Fast Mutagenesis System (Transgene biotech FM111-02) with full-length iASPP expressing plasmid as a template. All transfection experiments were conducted by using Lipofectamine 2000 (Invitrogen) following the manufacturer’s instructions.

### Western Blot (WB) assay

Cells were lysed in UREA buffer (8 M Urea, 1 M Thiourea, 0.5% CHAPS, 50 mM DTT, and 24 mM Spermine). Some amounts of protein lysates were subjected to SDS gel electrophoresis. The immune complex was detected by an ECL kit (Thermo Scientific). The v5 antibody was purchased from Bio-Rad(Cat: MCA1360). p53(Cat:60283-2-lg), NF-κBp65(Cat:10745-1-AP), myc(Cat:16286-1-AP), and loading control antibodies, GAPDH(Cat:60004-1-lg), α-tubulin(Cat:66031-1-lg), β-tubulin(Cat:10094-1-AP), and β-actin(Cat:66009-1-lg) antibodies were from Proteintech Ltd. p-p53 (Ser15) and p-NF-κBp65 (Ser536) antibody was brought from CST (Cat:12571, 3033). Bax and Bcl-2 were from Abclonal (cat: A0207 and A0208, respectively). Anti-iASPP antibody (LX49.3) was brought from Merck (cat: A4605).

### In vitro translation and immunoprecipitation

In vitro transcription and translation of plasmids were performed by using the Promega TNT T7 Quick coupled transcription/translation system (L1171, Promega) following the manufacturer’s instructions. The in vitro translated proteins were diluted in PBS. Immunoprecipitation was performed using ANTI-v5 (Bio-Rad). Equal amounts of lysate or IP complexes were resolved by 8% SDS-PAGE and then analyzed by WB assay.

### MTT

Cell proliferation rate was assessed by MTT assay. Briefly, MTT solution in 1×PBS was added to each well at the final concentration of 0.5 mg/ml. The plate was incubated for 3 h at 37 °C. The MTT medium was aspirated carefully and the dark-blue formazan was solubilized with DMSO. Optical density was measured with a spectrometer at 490 nm. Each experiment was conducted in triplicates and repeated independently 3 times.

### Luciferase reporter assay

Briefly, the same amounts PIG-3-, κB-, or p21-luciferase reporter together with either full length iASPP, iASPP(295–828) were transfected into the indicated cancer cells, respectively. Each of the transfections was included the same amount of Renilla, which was used to standardize transfection efficiency. Forty-eight hours after transfection, the luciferase activities in cell lysates were measured with the luciferase assay system (Promega). The relative luciferase reporter activity was normalized to the activity unit from the Renilla luciferase.

### Subcellular fractionation assay

Cytoplasm lysis buffer (10 mM HEPES pH 7.9, 10 mM KCl, 1.5 mM MgCl2, and 0.5 mM b-mercaptoethanol) was applied to cells, followed by moderate vortex for 15 s and 15–20 min incubation on ice. Additional 5 μl 10% NP-40 (Amersco) was then added to the mixture followed by another round of vortex and incubation. The cytoplasm fraction was obtained by collecting supernatant after centrifugation at 16,000x*g* for 10 min at 4 ˚C. The resulting pellet was lysed in the nuclear fraction buffer (10 mM HEPES pH 7.6, 1 mM DTT, 7.5 mM MgCl2, 0.2 mM EDTA, 0.3 M NaCl, 1 M UREA, and 1% NP-40). The supernatant was collected as the nuclear fraction by centrifugation at 16,000x*g* for 10 min at 4 ˚C.

### Apoptosis assay

After indicated treatments, both suspension and attached cells were collected gently. Cell density was adjusted to 5 × 10^6^ cells/ml. About 100 μl cell suspension was incubated with 2.5 μl AnnexinV/FITC for 10 min and then 2.5 μl PI (BD Pharmingen) for 5 min at room temperature in dark. The rate of apoptosis was measured by flow cytometry (BD Pharmingen).

### Chromatin immunoprecipitation (ChIP)

Chromatin immunoprecipitation (ChIP) assay was performed to estimate protein binding at promoter regions of the target genes. Briefly, 1 × 10^7^ cells were collected in lysis buffer A and B (buffer A: 5 mM PIPES, 85 mM KCl, and 0.5% NP40. buffer B: 1% SDS, 10 mM EDTA, and 50 mM Tris-HCl.) supplemented with Protease Inhibitor Cocktail and sonicated to generate chromatin samples with average fragment sizes of 100–500 bp. Cell lysates were treated with the indicated antibodies or IgG control at 4 °C overnight. Then, the supernatants were mixed with the blocked Protein A/D sepharose beads to collect the antibody-chromatin complexes. After washing four times in wash buffers (low salt buffer, high salt buffer, LiCl buffer, and TE buffer), the immunoprecipitated DNA was eluted and purified for the subsequent quantitative PCR (qPCR) analysis to detect relative enrichment of each TF on the indicated genes. The PCR reaction was performed at 58 °C for 35 cycles using 2×GoldStar Best MasterMix (Cwbiotech) with primers proximal to p53 or NF-κB binding sites at Bax and Bcl-2 promoters as follows (Bax promoter Forward: 5′-GGGTTATCTCTTGGGCTCACAA-3′, Bax promoter Reverse: 5′-GAGCTCTCCCCAGCGCA −3′, Bcl-2 promoter Forward: 5′-TACCCAGCCTCCGTTATCCT-3′, Bcl-2 promoter Reverse: 5′- CTTTGAGTTCGGTGGGGTCA −3′). The PCR product was visualized by 1% agarose gel electrophoresis. Enrichment in a specific site relative to appropriate input controls is shown in the bar graph (derived from three independent experiments). The antibodies used in this assay are listed below: p53 (Abcam), NF-κBp65 (CST), Goat Anti-Mouse IgG HRP (Abcam), and Goat Anti-Rabbit IgG HRP (Abcam).

### RNA extraction and quantitative RT-PCR

Total RNA was isolated with Trizol Reagent (Invitrogen) following the manufacture’s protocol and was subjected to reverse transcription with GoScriptTM reverse transcription system (Promega). The coprecipitated RNA quantitative real-time RT-PCR was performed in triplicate with an Applied Biosystems Prism 7500 Fast Sequence Detection System using TaqMan universal PCR master mix according to the manufacture’s protocol (Applied Biosystems Inc). Gene expression levels relative to GAPDH rRNA were calculated by a 2^−ΔΔCT^ method. The primer sequences used in RT-PCR were shown as follows (Bax Forward: 5′-TCCACCAAGAAGCTGAGCGAG-3′, Bax Reverse: 5′-GTCCAGCCCATGATGGTTCT-3′, Bcl-2 Forward: 5′- ATGTGTGTGGAGAGCGTCAA-3′, Bcl-2 Reverse: 5′-GGGCCGTACAGTTCCACAAA-3′, GAPDH Forward: 5′-CGACCACTTTGTCAAGCTCA-3′, GAPDH Reverse: 5′- ACTGAGTGTGGCAGGGACTC-3′).

### BrdU staining

BrdU incorporation assay was carried out by following the protocol provided by Cell Signaling Technology. Briefly, BrdU was diluted to a final concentration of 0.03 mg/mL with fresh DMEM and then applied onto the cells grown on slices. Cells were incubated with 1.5 M HCl followed by 5 min fixation in 70% cold ethanol. After 3% BSA block, Overnight immunostained with anti-BrdU antibody (CST) was then conducted as shown above. The antibody dilution ratio is 1 to 1000. The next day, the antibodies were washed three times with PBS, stained with fluorescent secondary antibody (Abcam), and incubated at room temperature for 1 h, and washed with PBS three times. The nucleus was visualized by DAPI staining. The representative images were captured by a Zeiss LSM510 confocal microscope (Carl Zeiss, Heidelberg, Germany)

### Statistical analysis

Statistical analysis was done by using the SPSS 21 software package. Data were presented as the means ± standard error of the means (SEM) or standard deviation (SD). Student *t*-test was applied to assess the statistical significance. *P* values <0.05 were considered significant.

## Supplementary information

Supplementary Figure legends

Supplementary Figure 1

Supplementary Figure 2

Supplementary Figure 3

Supplementary Figure 4

Supplementary Figure 5

## Data Availability

The datasets used and analyzed during the current study are available from the corresponding author on reasonable request.
