# Supplementary material for: Nuclear iASPP determines cell fate by selectively inhibiting either p53 or NF-κB
Source: Cell Death Discov. 2021 Jul 26;7:195. doi: 10.1038/s41420-021-00582-1 (PMC8313550; doi:10.1038/s41420-021-00582-1)
Supplement: Supplementary file 1 — Supplementary Figure legends [file 41420_2021_582_MOESM1_ESM.docx]

**Nuclear iASPP determines cell fate by selectively inhibiting either p53 or NF-κB**

Wenjie Ge1^,*^, Yudong Wang^1,*^, Shanliang Zheng^1^, Dong Zhao^1^, Xingwen Wang^1^, Xiaoshi Zhang^2,4^ ,Ying Hu^1,3,4^

^1^School of Life Science and Technology, Harbin Institute of Technology, Harbin, Heilongjiang Province, 150001, China

^2^Department of Clinical Laboratory, Qilu Hospital of Shandong University, Jinan, Shandong, China 250012

^3^Shenzhen Graduate School of Harbin Institute of Technology, Shenzhen, 518055, China

^4^To whom correspondence should be addressed: Ying Hu, Tel: +86-86403826, E-mail: [huying@hit.edu.cn](mailto:huying@hit.edu.cn); Xiaoshi Zhang, Tel: +86-18560083259, E-mail: [histone@126.com](mailto:histone@126.com)

* These authors contribute equally to this manuscript

**Supplementary Figure 1**

293T cells were transfected as indicated and treated with or without 5 μM 5-FU, then apoptosis levels were determined by Annexin V-FITC and PI staining. Relative protein levels were determined by western blot. GAPDH was used as an immunoblots loading control. **p*<0.05; ##*p*<0.01 relative to the 5-FU-treated Vector control; &*p*<0.05 relative to the 5-FU-treated FL control. N.S., not significant.

**Supplementary Figure 2**

(A) MCF-7 cells were transfected with iASPP plasmid and treated with or without 10 nM TNF-α. The subcellular localization of iASPP-V5 was determined by cell fractionation and western blot.

(B) MCF-7 cells were treated with DMSO, TNF-α and BAY, and cell proliferation was determined by BrdU incorporation assay. Scale bars 100 μm. The quantification of BrdU positive cells were shown in the bar graph. N.S., not significant.

**Supplementary Figure 3**

(A) 293T cells were transfected as indicated and treated with or without 5 μM 5-FU, then apoptosis levels were determined by Annexin V-FITC and PI staining. Relative protein levels were determined by western blot. GAPDH was used as an immunoblots loading control. **p*<0.05; &&*p*<0.01 relative to FL control. N.S., not significant.

(B) SK-BR3 cells were transfected with iASPP plasmid and then the subcellular localization of iASPP-V5 was determined by cell fractionation and western blot.

**Supplementary Figure 4**

(A,B) The transcriptional activities of NF-κBp65 and p53, as indicated by κB and PIG3 luciferase activity, respectively, were measured by a luciferase reporter assay in HCT116 cells (A). The levels of p-p53 and p-NF-κBp65 were determined by western blot. α-tubulin was used as a loading control (B). **p<0.01, ***p<0.001, N.S., not significant.

**Supplementary Figure 5**

(A) Expression of p300 was determined by western blot in MCF-7 and RKO cells. α-tubulin was used as an immunoblots loading control.

(B) HCT116 cells were transfected as indicated and treated with or without 1μg/mL Dox, the cell fractionation of iASPP was determined by western blot. Dox, doxorubicin.

(C) Expression of iASPP, LMNB1, NF-κBp65 and p53 was determined by western blot in HCT116 cells with the indicated treatments. α-tubulin was used as an immunoblots loading control.

(D) HCT116 cells were transfected as indicated and treated with or without Dox, then p53 and NF-κBp65 transcriptional activities, as indicated by p21 and κB luciferase activity, were measured by a luciferase reporter assay.

(E) Representative WB of iASPP and 80kda iASPP protein levels in 8 human colorectal cancer cell (T) and paired adjacent normal controls (N).
