# Supplementary figures and images for: Nuclear iASPP determines cell fate by selectively inhibiting either p53 or NF-κB
Source: Cell Death Discov. 2021 Jul 26;7:195. doi: 10.1038/s41420-021-00582-1 (PMC8313550; doi:10.1038/s41420-021-00582-1)

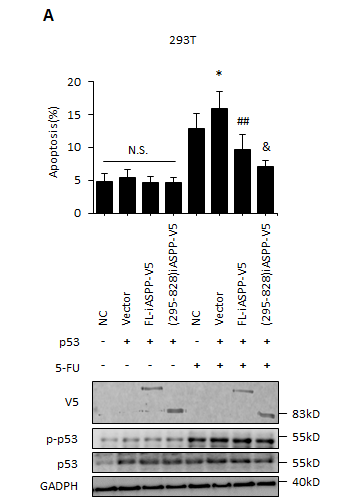

Supplement: Supplementary file 2 — Supplementary Figure 1 [file 41420_2021_582_MOESM2_ESM.tif]

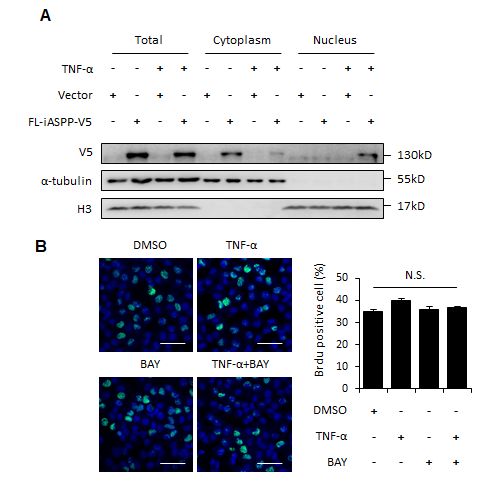

Supplement: Supplementary file 3 — Supplementary Figure 2 [file 41420_2021_582_MOESM3_ESM.tif]

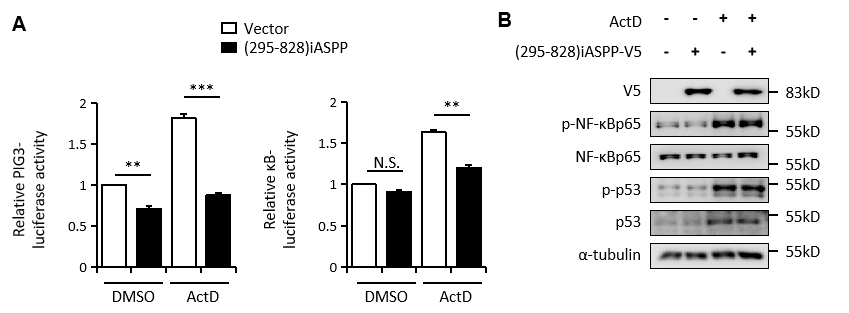

Supplement: Supplementary file 4 — Supplementary Figure 3 [file 41420_2021_582_MOESM4_ESM.tif]

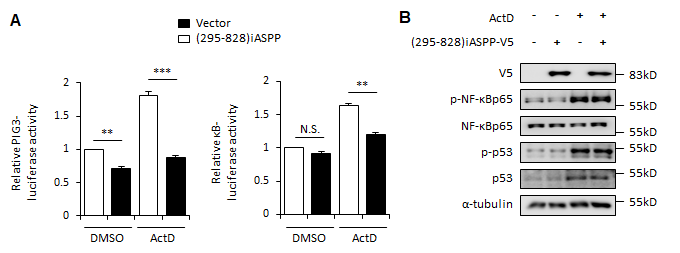

Supplement: Supplementary file 5 — Supplementary Figure 4 [file 41420_2021_582_MOESM5_ESM.tif]

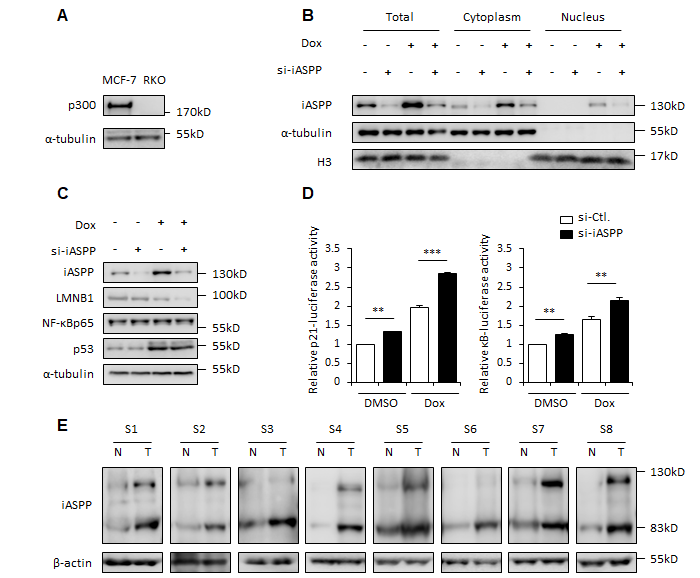

Supplement: Supplementary file 6 — Supplementary Figure 5 [file 41420_2021_582_MOESM6_ESM.tif]
